# 1-[(3-Nitro­phen­yl)(piperidin-1-yl)methyl]piperidine

**DOI:** 10.1107/S1600536812023525

**Published:** 2012-05-31

**Authors:** Zhe-Qin Wang, Yi Ma

**Affiliations:** a Center of Computers and Networks, Jilin Radio Television University, Jilin, Jilin Province 132001, People’s Republic of China; b Department of Chemical Engineering, Shandong Polytechnic University, Jinan 250353, People’s Republic of China

## Abstract

In the crystal structure of the title compound, C_17_H_25_N_3_O_2_, one-dimensional chains are formed *via* inter­molecular C—H⋯O hydrogen bonds along the *a* axis.

## Related literature

For the activities and uses of piperidine and its derivatives, see: Kumar *et al.* (2010 ▶); Huang *et al.* (2008 ▶); Cardellicchio *et al.* (2010 ▶); Wang *et al.* (2010 ▶).
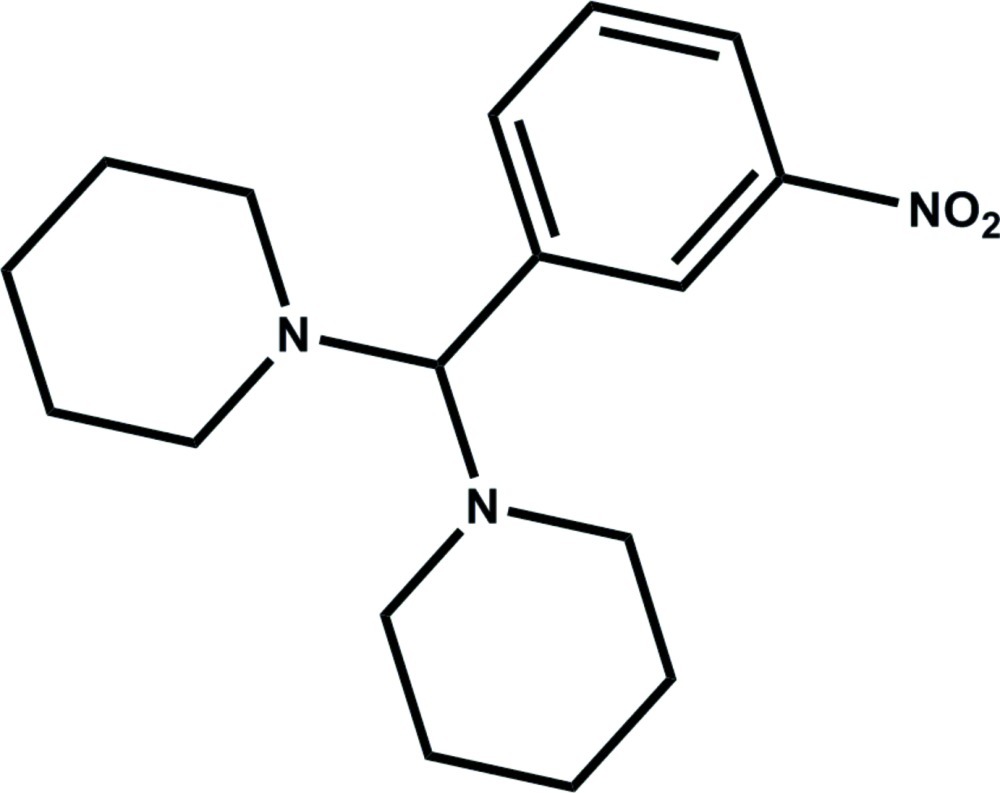


## Experimental

### 

#### Crystal data

C_17_H_25_N_3_O_2_
*M* *_r_* = 303.40
Orthorhombic, 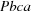

*a* = 12.1993 (14) Å
*b* = 8.2012 (9) Å
*c* = 33.453 (4) Å
*V* = 3347.0 (7) Å^3^
*Z* = 8
Mo *K*α radiation
μ = 0.08 mm^−1^
*T* = 296 K
0.40 × 0.20 × 0.20 mm

#### Data collection

Bruker SMART CCD area-detector diffractometer
Absorption correction: multi-scan (*SADABS*; Sheldrick, 1996 ▶) *T*_min_ = 0.981, *T*_max_ = 0.984
24143 measured reflections
3272 independent reflections
2633 reflections with *I* > 2σ(*I*)
*R*_int_ = 0.032

#### Refinement

*R*[*F* ^2^ > 2σ(*F* ^2^)] = 0.073
*wR*(*F* ^2^) = 0.231
*S* = 1.11
3272 reflections
199 parameters
H-atom parameters constrained
Δρ_max_ = 0.60 e Å^−3^
Δρ_min_ = −0.32 e Å^−3^

### 

Data collection: *SMART* (Bruker, 2001 ▶); cell refinement: *SAINT* (Bruker, 2001 ▶); data reduction: *SAINT*; program(s) used to solve structure: *SHELXS97* (Sheldrick, 2008 ▶); program(s) used to refine structure: *SHELXL97* (Sheldrick, 2008 ▶); molecular graphics: *SHELXTL* (Bruker, 2001 ▶); software used to prepare material for publication: *SHELXTL*.

## Supplementary Material

Crystal structure: contains datablock(s) global, I. DOI: 10.1107/S1600536812023525/aa2057sup1.cif

Structure factors: contains datablock(s) I. DOI: 10.1107/S1600536812023525/aa2057Isup2.hkl

Supplementary material file. DOI: 10.1107/S1600536812023525/aa2057Isup3.cml

Additional supplementary materials:  crystallographic information; 3D view; checkCIF report

## Figures and Tables

**Table 1:** Hydrogen-bond geometry (Å, °)

*D*—H⋯*A*	*D*—H	H⋯*A*	*D*⋯*A*	*D*—H⋯*A*
C5—H5*A*⋯O1^i^	0.93	2.43	3.332 (5)	165

Symmetry code: (i) 
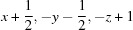
.

**Fig. 1.**
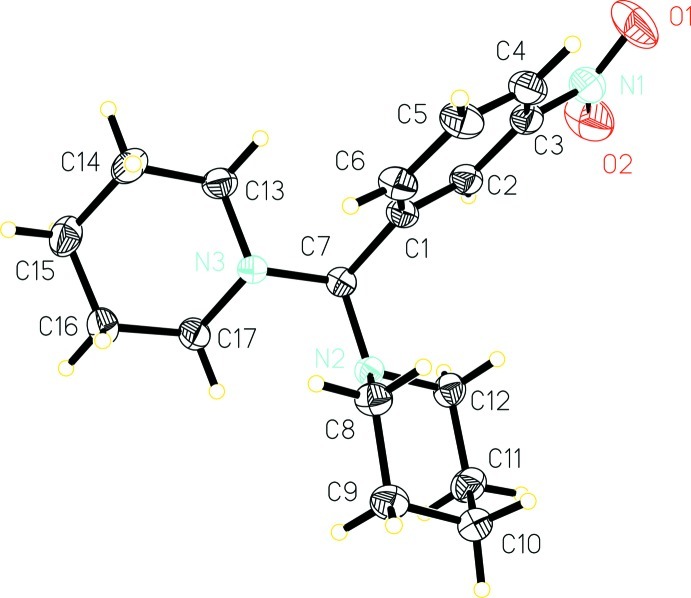
A drawing of the title compound, with the atom numbering scheme. Displacement ellipsoids are drawn at the 30% probability level.

**Fig. 2.**
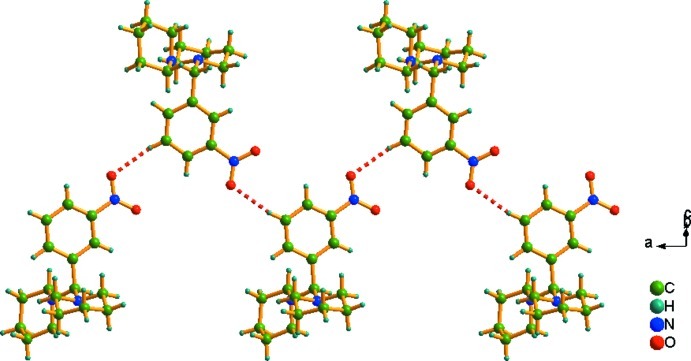
C—H···O interactions in the title compound.

## supplementary crystallographic information

### Comment

Piperidine and its derivatives extensively applied to some areas of bio-chemistry and material chemistry, which exhibit good bioactivities (Cardellicchio *et al.* 2010; Huang *et al.* 2008; Kumar *et al.* 2010). On the other hand, they display non-linear optic second harmonic generation response and ferroelectric properties (Wang *et al.*, 2010).

A view of the title structure is shown in Fig. 1. In the crystal structure, one-dimensional chains are formed *via* intermolecular C—H···O hydrogen bonds along the *a* axis (Table 1, Fig. 2).

### Experimental

1,1'-((3-nitrophenyl)methylene)dipiperidine (0.100 g) was dissolved in the mixed solvent containing ethanol (10 ml) and water (1 ml). The pale-yellow needle crystals suitable for X-ray diffraction were obtained after one week. Analysis found (%): C, 67.52; H, 8.33; N, 13.81%; calcd (%): C, 67.30; H, 8.31; N, 13.85%.

### Refinement

H atoms were calculated geometrically and refined as riding with C—H distances 0.93–0.97 Å and U_iso_(H) = 1.2 U_eq_(C)

### Crystal data

**Table:**

C_17_H_25_N_3_O_2_	*F*(000) = 1312
*M**_r_* = 303.40	*D*_x_ = 1.204 Mg m^−^^3^
Orthorhombic, *P**b**c**a*	Mo *K*α radiation, λ = 0.71073 Å
Hall symbol: -P 2ac 2ab	Cell parameters from 8288 reflections
*a* = 12.1993 (14) Å	θ = 2.5–27.3°
*b* = 8.2012 (9) Å	µ = 0.08 mm^−^^1^
*c* = 33.453 (4) Å	*T* = 296 K
*V* = 3347.0 (7) Å^3^	Needle, pale-yellow
*Z* = 8	0.40 × 0.20 × 0.20 mm

### Data collection

**Table:**

Bruker SMART CCD area-detector diffractometer	3272 independent reflections
Radiation source: fine-focus sealed tube	2633 reflections with *I* > 2σ(*I*)
Graphite monochromator	*R*_int_ = 0.032
φ and ω scans	θ_max_ = 26.0°, θ_min_ = 2.1°
Absorption correction: multi-scan (*SADABS*; Sheldrick, 1996)	*h* = −15→15
*T*_min_ = 0.981, *T*_max_ = 0.984	*k* = −9→10
24143 measured reflections	*l* = −41→40

### Refinement

**Table:**

Refinement on *F*^2^	Primary atom site location: structure-invariant direct methods
Least-squares matrix: full	Secondary atom site location: difference Fourier map
*R*[*F*^2^ > 2σ(*F*^2^)] = 0.073	Hydrogen site location: inferred from neighbouring sites
*wR*(*F*^2^) = 0.231	H-atom parameters constrained
*S* = 1.11	*w* = 1/[σ^2^(*F*_o_^2^) + (0.1025*P*)^2^ + 3.0609*P*] where *P* = (*F*_o_^2^ + 2*F*_c_^2^)/3
3272 reflections	(Δ/σ)_max_ < 0.001
199 parameters	Δρ_max_ = 0.60 e Å^−^^3^
0 restraints	Δρ_min_ = −0.32 e Å^−^^3^

### Special details

**Table:**

Geometry. All s.u.'s (except the s.u. in the dihedral angle between two l.s. planes) are estimated using the full covariance matrix. The cell s.u.'s are taken into account individually in the estimation of s.u.'s in distances, angles and torsion angles; correlations between s.u.'s in cell parameters are only used when they are defined by crystal symmetry. An approximate (isotropic) treatment of cell s.u.'s is used for estimating s.u.'s involving l.s. planes.
Refinement. Refinement of *F*^2^ against ALL reflections. The weighted *R*-factor *wR* and goodness of fit *S* are based on *F*^2^, conventional *R*-factors *R* are based on *F*, with *F* set to zero for negative *F*^2^. The threshold expression of *F*^2^ > σ(*F*^2^) is used only for calculating *R*-factors(gt) *etc*. and is not relevant to the choice of reflections for refinement. *R*-factors based on *F*^2^ are statistically about twice as large as those based on *F*, and *R*-factors based on ALL data will be even larger.

### Fractional atomic coordinates and isotropic or equivalent isotropic displacement parameters (Å^2^)

**Table:**

	*x*	*y*	*z*	*U*_iso_*/*U*_eq_	
C1	0.4378 (2)	0.0644 (3)	0.59215 (7)	0.0407 (6)	
C2	0.3469 (2)	0.0225 (3)	0.56963 (8)	0.0464 (7)	
H2A	0.2791	0.0697	0.5748	0.056*	
C3	0.3587 (2)	−0.0929 (4)	0.53875 (8)	0.0500 (7)	
C4	0.4570 (3)	−0.1650 (4)	0.53052 (9)	0.0555 (8)	
H4A	0.4625	−0.2418	0.5101	0.067*	
C5	0.5477 (3)	−0.1236 (4)	0.55250 (9)	0.0564 (8)	
H5A	0.6150	−0.1721	0.5471	0.068*	
C6	0.5383 (2)	−0.0096 (4)	0.58258 (8)	0.0493 (7)	
H6A	0.6005	0.0193	0.5970	0.059*	
C7	0.4282 (2)	0.1804 (3)	0.62769 (7)	0.0375 (6)	
H7A	0.3635	0.2493	0.6235	0.045*	
C8	0.5060 (2)	−0.0124 (4)	0.67719 (8)	0.0465 (7)	
H8A	0.5742	0.0475	0.6749	0.056*	
H8B	0.5092	−0.1051	0.6592	0.056*	
C9	0.4918 (3)	−0.0723 (4)	0.72010 (9)	0.0549 (8)	
H9A	0.5522	−0.1436	0.7271	0.066*	
H9B	0.4926	0.0200	0.7382	0.066*	
C10	0.3842 (3)	−0.1638 (4)	0.72440 (9)	0.0545 (7)	
H10A	0.3730	−0.1934	0.7522	0.065*	
H10B	0.3868	−0.2632	0.7087	0.065*	
C11	0.2898 (3)	−0.0577 (4)	0.71015 (9)	0.0566 (8)	
H11A	0.2225	−0.1205	0.7106	0.068*	
H11B	0.2811	0.0341	0.7282	0.068*	
C12	0.3100 (2)	0.0049 (4)	0.66804 (8)	0.0472 (7)	
H12A	0.3115	−0.0863	0.6496	0.057*	
H12B	0.2506	0.0767	0.6602	0.057*	
C13	0.5397 (3)	0.3828 (4)	0.59340 (8)	0.0496 (7)	
H13A	0.4815	0.4628	0.5911	0.060*	
H13B	0.5365	0.3119	0.5702	0.060*	
C14	0.6505 (3)	0.4692 (4)	0.59470 (9)	0.0549 (8)	
H14A	0.7087	0.3886	0.5955	0.066*	
H14B	0.6598	0.5337	0.5706	0.066*	
C15	0.6591 (3)	0.5790 (4)	0.63100 (10)	0.0592 (8)	
H15A	0.7333	0.6207	0.6332	0.071*	
H15B	0.6099	0.6710	0.6280	0.071*	
C16	0.6299 (3)	0.4858 (4)	0.66823 (9)	0.0581 (8)	
H16A	0.6256	0.5609	0.6906	0.070*	
H16B	0.6874	0.4076	0.6740	0.070*	
C17	0.5214 (2)	0.3963 (4)	0.66416 (8)	0.0489 (7)	
H17A	0.5070	0.3345	0.6883	0.059*	
H17B	0.4626	0.4747	0.6607	0.059*	
N1	0.2630 (3)	−0.1308 (4)	0.51498 (8)	0.0670 (8)	
N2	0.41423 (16)	0.0934 (3)	0.66589 (6)	0.0372 (5)	
N3	0.52421 (17)	0.2863 (3)	0.62994 (6)	0.0389 (5)	
O1	0.2761 (3)	−0.2159 (4)	0.48535 (9)	0.1021 (11)	
O2	0.1737 (2)	−0.0787 (5)	0.52516 (9)	0.1015 (11)	

### Atomic displacement parameters (Å^2^)

**Table:**

	*U*^11^	*U*^22^	*U*^33^	*U*^12^	*U*^13^	*U*^23^
C1	0.0456 (14)	0.0432 (14)	0.0334 (12)	−0.0001 (11)	−0.0022 (10)	−0.0005 (10)
C2	0.0489 (15)	0.0501 (15)	0.0401 (14)	−0.0021 (12)	−0.0025 (11)	0.0029 (12)
C3	0.0551 (16)	0.0565 (16)	0.0383 (14)	−0.0136 (14)	−0.0095 (12)	0.0089 (12)
C4	0.074 (2)	0.0512 (17)	0.0409 (15)	0.0023 (15)	0.0015 (14)	−0.0026 (13)
C5	0.0588 (18)	0.0623 (19)	0.0482 (16)	0.0130 (15)	0.0021 (13)	−0.0052 (14)
C6	0.0490 (15)	0.0576 (17)	0.0413 (14)	0.0055 (13)	0.0017 (12)	−0.0045 (12)
C7	0.0347 (12)	0.0407 (13)	0.0371 (13)	0.0028 (10)	0.0001 (9)	−0.0013 (10)
C8	0.0399 (14)	0.0556 (16)	0.0439 (15)	0.0035 (12)	−0.0019 (11)	0.0042 (12)
C9	0.0568 (17)	0.0593 (18)	0.0486 (16)	−0.0007 (14)	−0.0101 (13)	0.0069 (14)
C10	0.0681 (19)	0.0498 (16)	0.0455 (15)	−0.0089 (14)	−0.0024 (13)	0.0057 (12)
C11	0.0518 (16)	0.0615 (19)	0.0566 (17)	−0.0091 (14)	0.0087 (13)	0.0058 (15)
C12	0.0374 (14)	0.0520 (16)	0.0522 (16)	−0.0035 (12)	−0.0003 (11)	0.0023 (13)
C13	0.0599 (17)	0.0474 (15)	0.0415 (15)	0.0012 (13)	0.0030 (12)	0.0036 (12)
C14	0.0605 (18)	0.0465 (15)	0.0578 (18)	−0.0044 (14)	0.0179 (14)	0.0055 (13)
C15	0.0619 (18)	0.0430 (15)	0.073 (2)	−0.0094 (14)	0.0103 (15)	−0.0014 (14)
C16	0.0659 (19)	0.0538 (17)	0.0547 (17)	−0.0154 (15)	0.0014 (14)	−0.0110 (14)
C17	0.0556 (16)	0.0473 (15)	0.0436 (15)	−0.0056 (13)	0.0081 (12)	−0.0091 (12)
N1	0.0709 (19)	0.077 (2)	0.0527 (15)	−0.0188 (16)	−0.0139 (14)	0.0004 (14)
N2	0.0347 (11)	0.0395 (11)	0.0373 (11)	−0.0002 (9)	0.0013 (8)	−0.0005 (9)
N3	0.0419 (11)	0.0413 (12)	0.0335 (10)	−0.0029 (9)	0.0036 (8)	−0.0016 (8)
O1	0.105 (2)	0.126 (3)	0.0762 (17)	−0.013 (2)	−0.0286 (16)	−0.0419 (19)
O2	0.0589 (16)	0.154 (3)	0.091 (2)	−0.0134 (18)	−0.0165 (14)	−0.022 (2)

### Geometric parameters (Å, º)

**Table:**

C1—C2	1.384 (4)	C11—C12	1.519 (4)
C1—C6	1.405 (4)	C11—H11A	0.9700
C1—C7	1.527 (3)	C11—H11B	0.9700
C2—C3	1.408 (4)	C12—N2	1.466 (3)
C2—H2A	0.9300	C12—H12A	0.9700
C3—C4	1.366 (4)	C12—H12B	0.9700
C3—N1	1.447 (4)	C13—N3	1.469 (3)
C4—C5	1.371 (5)	C13—C14	1.527 (4)
C4—H4A	0.9300	C13—H13A	0.9700
C5—C6	1.378 (4)	C13—H13B	0.9700
C5—H5A	0.9300	C14—C15	1.515 (4)
C6—H6A	0.9300	C14—H14A	0.9700
C7—N3	1.460 (3)	C14—H14B	0.9700
C7—N2	1.473 (3)	C15—C16	1.504 (4)
C7—H7A	0.9800	C15—H15A	0.9700
C8—N2	1.466 (3)	C15—H15B	0.9700
C8—C9	1.527 (4)	C16—C17	1.520 (4)
C8—H8A	0.9700	C16—H16A	0.9700
C8—H8B	0.9700	C16—H16B	0.9700
C9—C10	1.518 (4)	C17—N3	1.458 (3)
C9—H9A	0.9700	C17—H17A	0.9700
C9—H9B	0.9700	C17—H17B	0.9700
C10—C11	1.521 (4)	N1—O2	1.219 (4)
C10—H10A	0.9700	N1—O1	1.223 (4)
C10—H10B	0.9700		
			
C2—C1—C6	117.9 (2)	H11A—C11—H11B	108.0
C2—C1—C7	121.1 (2)	N2—C12—C11	110.7 (2)
C6—C1—C7	120.9 (2)	N2—C12—H12A	109.5
C1—C2—C3	119.0 (3)	C11—C12—H12A	109.5
C1—C2—H2A	120.5	N2—C12—H12B	109.5
C3—C2—H2A	120.5	C11—C12—H12B	109.5
C4—C3—C2	121.9 (3)	H12A—C12—H12B	108.1
C4—C3—N1	120.3 (3)	N3—C13—C14	109.9 (2)
C2—C3—N1	117.7 (3)	N3—C13—H13A	109.7
C3—C4—C5	119.5 (3)	C14—C13—H13A	109.7
C3—C4—H4A	120.2	N3—C13—H13B	109.7
C5—C4—H4A	120.2	C14—C13—H13B	109.7
C4—C5—C6	119.5 (3)	H13A—C13—H13B	108.2
C4—C5—H5A	120.2	C15—C14—C13	111.1 (2)
C6—C5—H5A	120.2	C15—C14—H14A	109.4
C5—C6—C1	122.1 (3)	C13—C14—H14A	109.4
C5—C6—H6A	118.9	C15—C14—H14B	109.4
C1—C6—H6A	118.9	C13—C14—H14B	109.4
N3—C7—N2	109.63 (19)	H14A—C14—H14B	108.0
N3—C7—C1	110.41 (19)	C16—C15—C14	110.2 (2)
N2—C7—C1	112.5 (2)	C16—C15—H15A	109.6
N3—C7—H7A	108.1	C14—C15—H15A	109.6
N2—C7—H7A	108.1	C16—C15—H15B	109.6
C1—C7—H7A	108.1	C14—C15—H15B	109.6
N2—C8—C9	110.3 (2)	H15A—C15—H15B	108.1
N2—C8—H8A	109.6	C15—C16—C17	112.2 (3)
C9—C8—H8A	109.6	C15—C16—H16A	109.2
N2—C8—H8B	109.6	C17—C16—H16A	109.2
C9—C8—H8B	109.6	C15—C16—H16B	109.2
H8A—C8—H8B	108.1	C17—C16—H16B	109.2
C10—C9—C8	110.3 (2)	H16A—C16—H16B	107.9
C10—C9—H9A	109.6	N3—C17—C16	110.4 (2)
C8—C9—H9A	109.6	N3—C17—H17A	109.6
C10—C9—H9B	109.6	C16—C17—H17A	109.6
C8—C9—H9B	109.6	N3—C17—H17B	109.6
H9A—C9—H9B	108.1	C16—C17—H17B	109.6
C9—C10—C11	110.0 (2)	H17A—C17—H17B	108.1
C9—C10—H10A	109.7	O2—N1—O1	123.0 (3)
C11—C10—H10A	109.7	O2—N1—C3	119.5 (3)
C9—C10—H10B	109.7	O1—N1—C3	117.5 (3)
C11—C10—H10B	109.7	C8—N2—C12	110.9 (2)
H10A—C10—H10B	108.2	C8—N2—C7	114.97 (19)
C12—C11—C10	111.2 (2)	C12—N2—C7	112.48 (19)
C12—C11—H11A	109.4	C17—N3—C7	112.92 (19)
C10—C11—H11A	109.4	C17—N3—C13	108.8 (2)
C12—C11—H11B	109.4	C7—N3—C13	112.4 (2)
C10—C11—H11B	109.4		

### Hydrogen-bond geometry (Å, º)

**Table:**

*D*—H···*A*	*D*—H	H···*A*	*D*···*A*	*D*—H···*A*
C5—H5*A*···O1^i^	0.93	2.43	3.332 (5)	165

Symmetry code: (i) *x*+1/2, −*y*−1/2, −*z*+1.
